# Stage IV uterine leiomyosarcoma resection: A case report

**DOI:** 10.1016/j.amsu.2022.103905

**Published:** 2022-06-08

**Authors:** Mohammad Badr Almoshantaf, Talal Orabi, Weaam Ezzdean, Mahmoud Karnabeh

**Affiliations:** a Neurosurgery Department, Ibn Al Nafees Hospital, Damascus, Syria; b General Surgery Department, Ibn Al Nafees Hospital, Damascus, Syria; c Urological Department, Ibn Al Nafees Hospital, Damascus, Syria

**Keywords:** Leiomyosarcoma, Uterine, Stage IV, Surgery

## Abstract

We present a case of stage IV uterine leiomyosarcoma that was treated with total hysterectomy, bilateral salpingo-oophorectomy, and extensive resection of a 14 kg abdominal mass, as well as complete eradication of accompanying symptoms. This case may prompt researchers to look for other surgical solutions to similar issues.

## Highlights

• Stage IV Uterine Leiomyosarcoma can be surgically curable.
• Total Hysterectomy and Bilateral Salpingo-Oophorectomy is the surgical choice in massive Uterine Leiomyosarcoma.
• Post-operation Follow-ups with Ultrasonography at a 3-months intervals are crucial to detect early relapse.

## 1 Introduction

Leiomyosarcoma (LMS) is a malignant mesenchymal tumor that arises from smooth muscle cells or mesenchymal stem cells. LMS accounts for about a quarter of all soft tissue sarcomas, and the incidence depends on gender, subtype, and location [1]. Theoretically, LMS can occur in any soft tissue throughout the body but is most common in the uterus and posterior peritoneum [2]. Uterine leiomyosarcoma (ULMS) is rare but is considered to be the most common uterine sarcoma [3]. The exact cause of ULMS is unknown, but several associations have been reported in the literature, including Epstein-Barr virus (EBV) infection, acquired immunodeficiency syndrome (AIDS), and organ transplantation [4].

LMS staging and grading systems are commonly used to create risk profiles. However, ULMS itself contributes to a poor prognosis, even if it is confined to the uterus. Currently, the revised criteria of the International Association of Obstetrics and Gynecology (FIGO) is superior to other classification systems in terms of progression and overall survival but is not ideal [5,6]. ULMS symptoms depend on the exact location, size, and course. Although it may be asymptomatic, the most common symptom is abnormal bleeding from the uterus, and postmenopausal bleeding is an important indicator of ULMS. Other symptoms include pressure and pain in the pelvis and stomach, changes in bladder and bowel habits, and common cancer symptoms. Both normal signs and pathological features in diagnostic imaging have made diagnosis possible only by histological examination of tumor specimens after surgery [1].

Surgery is the main treatment option. In general, aggressive surgical cytoreduction at the time of initial diagnosis offers the possibility of prolonging survival or healing [7]. Initial treatment for LMS includes removal by hysterectomy However, bilateral salpingo-oophorectomy is controversial [8]. In addition, non-surgical and recurrent cases can benefit from complementary chemotherapy and high-intensity focused ultrasound [9].

We present a case of high-grade stage IV ULMS. Hysterectomy and bilateral salpingo-oophorectomy were performed and 14 kg of abdominal masses were resected. It was then followed by subtotal resolution of the tumor's symptoms The manuscript has been reported in line with the SCARE 2020 criteria [10].

## 2 Case report/case presentation

We present a 53-years-old multiparous nonsmoker female.. Her pharmaceutical, familial, and medical histories were all uncomplicated. Her surgical history included only two cesarean sections.

She presented to her local clinic two years ago with vague abdominal and lumbar pain. Her pain was unrelated to posture or dietary habits. No aggravating or alleviating factors were reported.

During clinical examination, mild discomfort was noted in the left upper quadrant during palpation. Routine blood work and U&E were within normal limits. An abdominal ultrasound revealed a mass in the left upper quadrant and some irregularities in the abdomen, but without proper details. CT imaging has been ordered and the results were:

### 2.1 Chest

Observation of (7*10) cm mass in the medial basal left chest. Observation of small multiple bilateral masses in the lung parenchyma. Observation of minimal bilateral pleural effusion.

### 2.2 Abdomen and pelvis

Observation of wide-spread multiple necrotic lesions; the biggest of those measures (25*30) cm.

Later, a needle biopsy of the chest mass has been ordered. Microscopic pathology revealed the presence of a few macrophages with focal small alveoli, fibrotic walls, chronic inflammatory infiltrate, and anthracosis. This pathology was consistent with hepatic cell carcinoma, but immunostaining showed Chromogranin positivity and on this basis, a diagnosis of the moderately differentiated neuroendocrine tumor was made. As a result, it was initially placed on Octreotide.

After 4 months, MSCT was ordered for follow-up. It results in slight development of the tumor as follows:

### 2.3 Chest

**Fig. 1:**
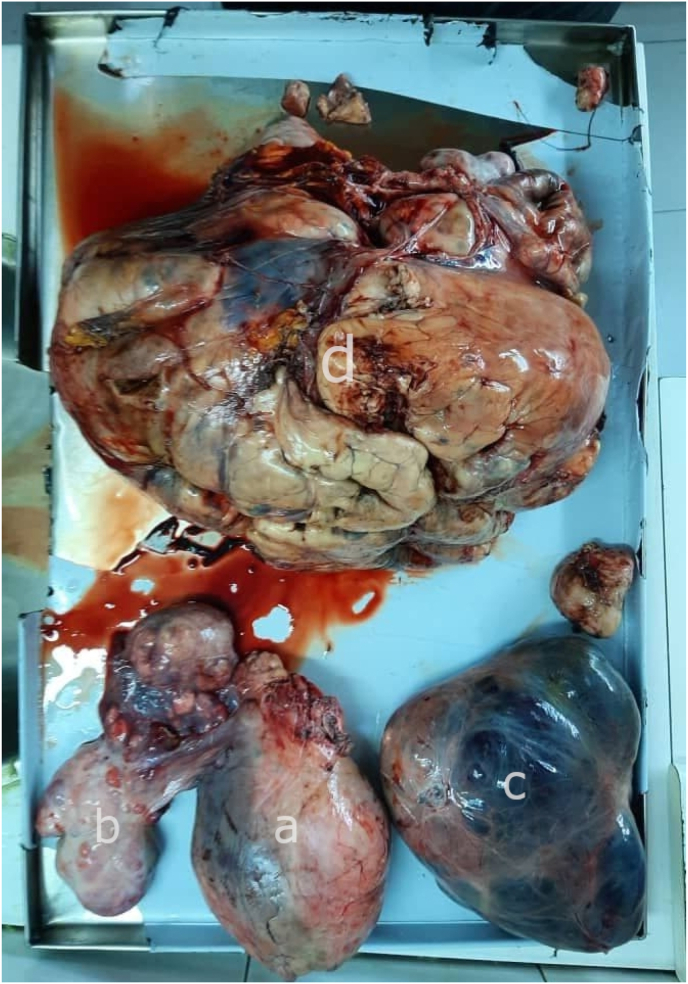
Uterine, Left Ovary, Right Ovary, Abdomen mass (35*25) cm, 14 kg.

The (7*10) cm mass in the medial basal left chest has started stretching to the posterior mediastinum. The multiple bilateral masses showed no signs of retraction. Lymphadenopathy in the mediastinum and the axilla (see Fig. 1).

### 2.4 Abdomen and pelvis

A huge, space-occupying mass begins to march up from the pelvis until it reaches the diaphragm. It's pushing relevant relationships through its course. It includes macrocalcifications and multinecrotic liquefied hypoechoic areas (shown in Fig. 3 and Fig. 4). Lymphadenopathy in para-aortic nodes.

**Fig. 2:**
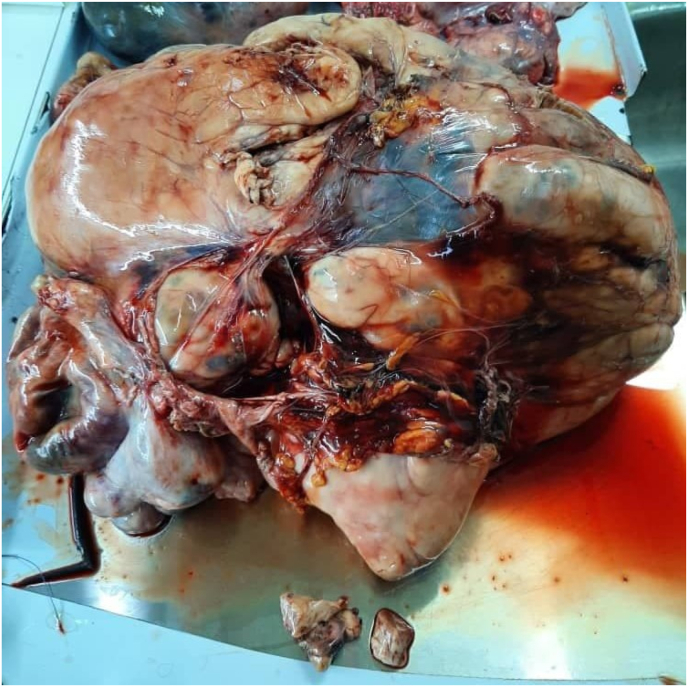
Abdomen mass (35*25) cm, 14 kg.

**Fig. 3:**
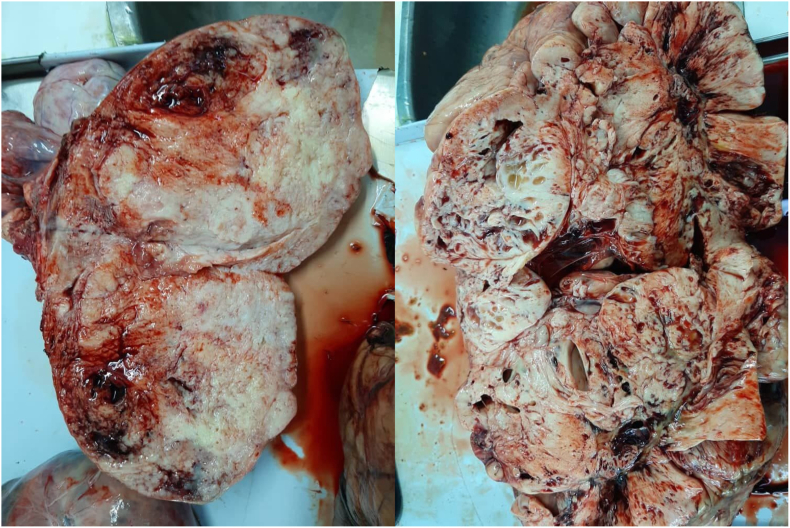
Dissected abdomen mass (35*25) cm, 14 kg.

**Fig. 4:**
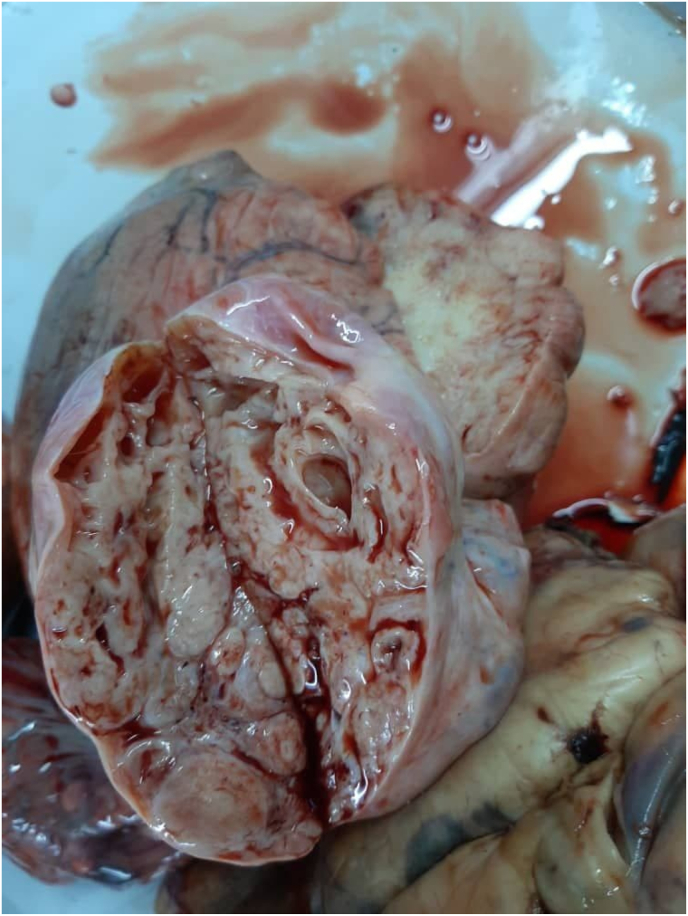
Dissected uterine.

At this point, the patient has unintentionally lost more than 15% of their body weight since the first presentation. She reported that her appetite was significantly reduced due to vague general abdominal pain and persistent vomiting. She also suffered from constipation and painful bowel movements. After several discussions with general surgery, she preferred to forgo the palliative solution and to perform an excisional operation. Based on this, elective laparotomy was planned.

After proper examination of the abdomen and pelvis, total hysterectomy and bilateral salpingo-oophorectomy were decided. In addition, a total of 14 kg of mass was removed from the abdomen, allowing decompression of the small intestine, large intestine, stomach, and kidneys. Lung lesions were left without intervention (Shown in Fig. 2). Pathology concluded high-grade leiomyosarcoma with predominant epithelial cell patterns with free tumor margins. Diffuse positive actin markers were noted.

After more than a year of surgical resection, she reports happiness with full resolution of abdominal symptoms. She has regained her weight. She is on multiple courses of Gemcitabine and Vinorelbine. Her follow-up b laboratory tests show no abnormal findings other than occasional leukocytopenia that can be explained by chemotherapy. According to follow-up CT, lung lesions are showing small or unnoticeable retraction. No respiratory symptoms are reported at the moment.

## 3 Discussion/conclusion

This ULMS is a stage IVB according to FIGO. Some conservative surgical treatment is offered, yet it's not traditionally treated with wide excision surgery. However, nothing in the literature -to the extent of our knowledge-goes completely against it.

The main purpose of excisional surgery was to decompress the abdominal structures. The purpose was achieved as she regained her weight with normal eating and bowel movements. The uterus played an obscure role in the lesion prior to surgery.MRI was not possible at the time due to the lack of hospital equipment. Total hysterectomy and bilateral salpingo-oophorectomy were decided after intraoperative inspection of the uterus and ovaries. We concluded that the primary tumor was ULMS. The metastases and the deposits in the abdomen were likely the cause of the first complaints of abdominal pain. A later resection operation for lung metastases should be planned.

It is known that uterine sarcomas spread via both lymphatic and hematogenous dissemination. Similar to our case, distant metastasis to the lung can frequently happen. Ovarian metastasis is considered rare [11]. In Fig,1, C, ovarian cysts which can be seen with macroscopic capsular involvement are most likely to be malignant cysts due to the hematogenous spread of ULMS.

Ultrasonography is being repeated at a 3-months interval as it’s more reliable for postoperative follow-up than preoperative diagnosis [12]. No recurrence is noted. We are planning to keep close monitoring her for 3 years postoperatively as recurrence is common in this time frame.

We do not know whether the premenopausal status causes a good prognosis because the postmenopausal status is associated with histological types of poor prognosis [13]. No abnormal uterine bleeding has been reported. Compression-related gastrointestinal symptoms were the highlighted symptoms. No neoadjuvant chemotherapy was ordered. ULMS is not known to be chromogranin positive, so it is most likely a false positive. Needless to say, octreotide had no noticeable effect on the tumor.

This case shows adequate surgical resolution of the Stage IV ULMS primary tumor. Although it is common practice for Stage IV cancer not to be treated surgically, we recommend further studies along with this case to discuss and revise current guidelines.

## Statement of ethics

The case report conducted ethically in accordance with the World Medical Association Declaration of Helsinki. The study was approved by Ibn Al Nafees Hospital Committee, decision number N1178 dated 04/05/2021. The patient has given their written informed consent to publish their case (including publication of images).

## Funding sources

This research did not receive any specific grant from funding agencies in the public, commercial, or not-for-profit sectors.

## Author contributions

All authors have participated in writing and reviewing the manuscript.

## Registration of research studies

Not applicable.

## Guarantor

Mohammad Badr Almoshantaf.

## Data availability statement

The data that support this case report is not publicly available due to privacy purposes but are available from the corresponding author upon reasonable request.

## Provenance and peer review

Not commissioned, externally peer-reviewed.

## Patient consent

Written informed consent was obtained from the patient for publication of this case report and accompanying images. A copy of the written consent is available for review by the Editor-in-Chief of this journal on request.

## Declaration of competing interest

The authors have no conflicts of interest to declare.
